# Room-Temperature Assembled MXene-Based Aerogels for High Mass-Loading Sodium-Ion Storage

**DOI:** 10.1007/s40820-021-00781-6

**Published:** 2021-12-17

**Authors:** Fei Song, Jian Hu, Guohao Li, Jie Wang, Shuijiao Chen, Xiuqiang Xie, Zhenjun Wu, Nan Zhang

**Affiliations:** 1 College of Materials Science and Engineering, Hunan University, Changsha, 410082 People’s Republic of China; 2 College of Chemistry and Chemical Engineering, Hunan University, Changsha, 410082 People’s Republic of China

**Keywords:** MXenes, Aerogel, Room-temperature assembly, Interfacial engineering, Sodium-ion storage

## Abstract

Low-temperature assembly of MXene nanosheets into three-dimensional (3D) robust aerogels addresses the crucial stability concern of the nano-building blocks during the fabrication process, which is of key importance for transforming the fascinating properties at the nanoscale into the macroscopic scale for practical applications. Herein, suitable cross-linking agents (amino-propyltriethoxysilane, Mn^2+^, Fe^2+^, Zn^2+^, and Co^2+^) as interfacial mediators to engineer the interlayer interactions are reported to realize the graphene oxide (GO)-assisted assembly of Ti_3_C_2_T_*x*_ MXene aerogel at room temperature. This elaborate aerogel construction not only suppresses the oxidation degradation of Ti_3_C_2_T_*x*_ but also generates porous aerogels with a high Ti_3_C_2_T_*x*_ content (87 wt%) and robustness, thereby guaranteeing the functional accessibility of Ti_3_C_2_T_*x*_ nanosheets and operational reliability as integrated functional materials. In combination with a further sulfur modification, the Ti_3_C_2_T_*x*_ aerogel electrode shows promising electrochemical performances as the freestanding anode for sodium-ion storage. Even at an ultrahigh loading mass of 12.3 mg cm^−2^, a pronounced areal capacity of 1.26 mAh cm^−2^ at a current density of 0.1 A g^−1^ has been achieved, which is of practical significance. This work conceptually suggests a new way to exert the utmost surface functionalities of MXenes in 3D monolithic form and can be an inspiring scaffold to promote the application of MXenes in different areas.

**Supplementary Information:**

The online version contains supplementary material available at 10.1007/s40820-021-00781-6.

## Highlights

- Room temperature graphene oxide-assisted assembly of 3D Ti_3_C_2_T_*x*_ MXene aerogels have been realized by introducing interfacial mediators (amino-propyltriethoxysilane, Mn^2+^, Fe^2+^, Zn^2+^, and Co^2+^).
- The methodology not only suppresses the oxidation degradation of Ti_3_C_2_T_*x*_, but also generates porous aerogels with a high Ti_3_C_2_T_*x*_ content (87 wt%) and robustness.
- As freestanding electrode of the as-prepared Ti_3_C_2_T_*x*_-based aerogel with a practical-level mass loading of 12.3 mg cm^-2^ still delivers an areal capacity of 1.26 mAh cm^-2^ at a current density of 0.1 A g^-1^.

**Supplementary Information:**

The online version contains supplementary material available at 10.1007/s40820-021-00781-6.

## Graphical abstract

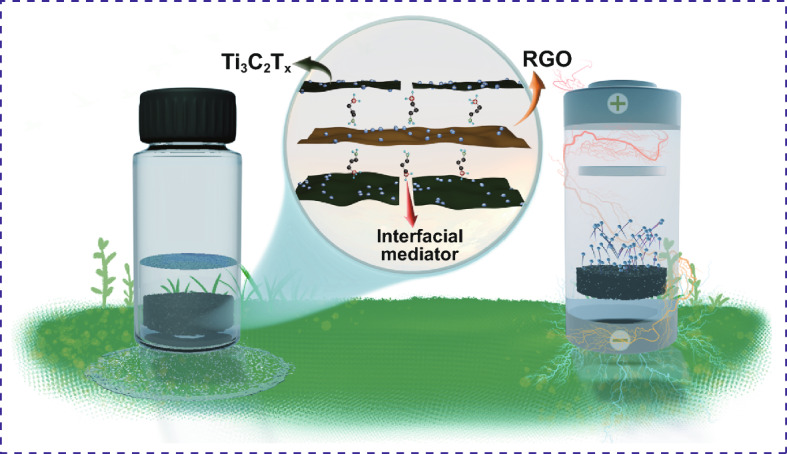

## Introduction

MXenes, an emerging family of two-dimensional (2D) transition metal carbides and nitrides, have aroused extensive research in many fields [1, 2]. Tens of MXenes have been synthesized and extensively studied, showing promising prospects [3]. As a representative MXene, Ti_3_C_2_T_*x*_ (where T_*x*_ is surface terminations of –OH, –O, and –F) is the most widely studied [4–7]. Sodium-ion-based energy storage system has become a candidate for the next-generation energy storage owing to its low cost and abundant resources [8–10]. Ti_3_C_2_T_*x*_ shows great potential for electrochemical lithium-ion, sodium-ion and potassium-ion storage [11–20]. Typically, Ti_3_C_2_T_*x*_ possesses favorable adsorption [16] energies that fit with the sodium-ion storage [21, 22]. Nevertheless, 2D Ti_3_C_2_T_*x*_ nanosheets are prone to restack, which limits the performance and application of the few-layered Ti_3_C_2_T_*x*_. Especially, it is still a challenge to scale the electrochemical performances of Ti_3_C_2_T_*x*_ to electrodes with practical-level mass loadings (> 10 mg cm^−2^) due to the limited ion diffusion in thicker electrodes [23].

Three-dimensional (3D) Ti_3_C_2_T_*x*_ with favorable surface accessibility represents a suitable integrated functional infrastructure for practical applications, exhibiting promising performances in emerging fields such as electrochemical energy storage and electromagnetic shielding [24–26]. The 3D open structure facilitates faster ion transport and higher surface utilization. Notably, the mutually repulsive hydrophilic groups (–OH, –O, etc.) on the surface of Ti_3_C_2_T_*x*_ make it hard for the cross-linking of adjacent Ti_3_C_2_T_*x*_ nanosheets directly [27, 28]. Divalent metal ions have been applied as the initiator for the gelation of Ti_3_C_2_T_*x*_ colloidal suspension [29]. However, the dissolved free metal ions have limited selectivity for the 3D interconnection of Ti_3_C_2_T_*x*_ nanosheets, which also lead to the face-to-face restacking of Ti_3_C_2_T_*x*_, thereby resulting in a less developed interconnected porous structure. Graphene oxide (GO)-assisted assembly of Ti_3_C_2_T_*x*_ nanosheets has been widely explored, which relies critically on the construction of 3D reduced graphene oxide (RGO) scaffold by balancing the hydrophilicity−hydrophobicity during the reduction of GO. It is worth noting that most of the reported GO-assisted 3D assembly of Ti_3_C_2_T_*x*_ is carried out at elevated temperatures to afford proper interactions between RGO and Ti_3_C_2_T_*x*_ while overcoming their electrostatic repulsions (Table S1) [30–33]. This raises the concern of Ti_3_C_2_T_*x*_ oxidation degradation due to the exposure of thermodynamically metastable marginal transition metal atoms, which sacrifices the appealing properties of Ti_3_C_2_T_*x*_ [34]. For room temperature GO-assisted 3D assembly of Ti_3_C_2_T_*x*_, the construction of monolithic structures with a relatively low ratio of Ti_3_C_2_T_*x*_ (≤ 70 wt%) has been successful, but only aggregated powders can be produced at a higher Ti_3_C_2_T_*x*_ ratio (Table S1) [35]. The construction of 3D monolithic aerogels with a high Ti_3_C_2_T_*x*_ ratio by GO-assisted assembly at room temperature is highly desirable to exert the potential of Ti_3_C_2_T_*x*_ for macroscale applications but remains a challenge. Zhang et al. reviewed the characteristics, treatment methods, interfacial assembly, and applications of MXene colloidal solutions, which provided guidelines for the subsequent study of MXene [36–39].

Noteworthily, the dispersing effect of Ti_3_C_2_T_*x*_ originating from the electrostatic repulsion between Ti_3_C_2_T_*x*_ and RGO becomes dominant for the room temperature GO-assisted assembly at a high Ti_3_C_2_T_*x*_ ratio, which is the key factor destroying the continuous anisotropic intersheet cross-linking of 3D RGO to form integrated structures and thus the formation of Ti_3_C_2_T_*x*_/RGO monoliths. Therefore, our particular interest focuses on the modulation of the interfacial chemistry between Ti_3_C_2_T_*x*_ and RGO to develop methods for the controllable and efficient assembly of Ti_3_C_2_T_*x*_ into functional monoliths at room temperature. In this work, suitable cross-linking agents (amino-propyltriethoxysilane, Mn^2+^, Fe^2+^, Zn^2+^, and Co^2+^) as interfacial mediators have been used to reduce the electrostatic repulsion between electronegative interfaces of Ti_3_C_2_T_*x*_ and GO to construct Ti_3_C_2_T_*x*_/RGO hydrogels at room temperature. After freeze-drying, the obtained Ti_3_C_2_T_*x*_/RGO composite aerogels possess excellent structural robustness even at a high Ti_3_C_2_T_*x*_ ratio of 87 wt%, which renders the Ti_3_C_2_T_*x*_/RGO composite aerogel a potential candidate for fabricating self-supporting electrodes for electrochemical sodium storage. When Ti_3_C_2_T_*x*_/RGO composite aerogels are applied to electrochemical energy storage, we refer to the sulfur template and surface modification methods reported in the literature to improve the electrochemical performance of the material [40–42]. On the basis of the developed strategy, sulfur modification has been further performed by using elemental sulfur/Ti_3_C_2_T_*x*_ composites (S/Ti_3_C_2_T_*x*_) as the starting material followed by thermal treatment to prepare the sulfur-doped Ti_3_C_2_T_*x*_/RGO aerogels for electrochemical sodium storage. Encouragingly, benefiting from the enhanced charge transfer kinetics due to the favorable porous structure and sulfur modification, a pronounced areal capacity of 1.26 mAh cm^−2^ at a current density of 0.1 A g^−1^ has been achieved at a high loading mass of 12.3 mg cm^−2^, demonstrating great promise for practical applications.

## Experimental

### Preparation of Ti_3_C_2_T_***x***_ and GO

Ti_3_C_2_T_*x*_ colloid suspension was synthesized by selectively etching Al elements from the Ti_3_AlC_2_ MAX phase with LiF/HCl [43, 44]. Briefly, 3.2 g of LiF was dissolved in 40 mL (9 M) HCl solution and stirred uniformly in a Teflon reactor. Then, 2 g of Ti_3_AlC_2_ powder was steadily added to the above solution, followed by magnetic stirring at 35 °C for 24 h in a fume hood. After that, the obtained slurry was washed to neutral with deionized water by repeated centrifugation and at 7000 rpm. Next, deionized water was added to the Ti_3_C_2_T_*x*_ slurry and the ultrasonic treatment was applied under the protection of N_2_. Finally, the Ti_3_C_2_T_*x*_ colloid suspension was obtained after centrifugation at 3500 rpm for 1 h. GO was synthesized from natural graphite powder according to the modified Hummers method [45].

### Assembly of MGA

A 60 mg Ti_3_C_2_T_*x*_ powder obtained by freeze-drying above Ti_3_C_2_T_*x*_ colloid suspension and 1 mL of GO solution (10 mg mL^−1^) were mixed and ground in a mortar for minutes until a uniform black slurry formed. Subsequently, 10 μL of APTES was added to the mixed slurry as an interfacial mediator, and further stirring was performed for uniform mixing. After that, 4 mL of VC solution (0.25 g mL^−1^) was added to the mixed slurry and stirred for another 20 min. Thereafter, the mixture was immediately transferred and sealed into a 20 mL glass vial for slow reduction at 25 °C for 72 h. A black Ti_3_C_2_T_*x*_/RGO hydrogel gradually formed, and the solution became yellow. After the reaction, the yellow solution was poured out mildly, and the hydrogel was washed with deionized water to remove excess reactants and impurities. The Ti_3_C_2_T_*x*_/RGO aerogels were obtained by freeze-drying the above Ti_3_C_2_T_*x*_/RGO hydrogel and sintering in a tube furnace at 400 °C under N_2_ for 4 h. Both deionized water washing and heat-treatment help to remove VC residues. In a similar way, APTES was replaced with 10 mg of metal ions salts (MnCl_2_, FeCl_2_, ZnCl_2_, and Co(CH_3_COO)_2_) dissolved in 0.5 mL of deionized water to obtain Ti_3_C_2_T_*x*_/RGO hydrogels cross-linked by different metal ions. To alleviate the oxidation induced by these metal ions, N_2_ bubbling was additionally applied.

### Assembly of SMGA

Ti_3_C_2_T_*x*_@S composites were prepared using a modification of the method reported by Tang et al. [46]. Sodium polysulfides (NaPS) was selected as a suitable sulfur source. Specifically, 0.4 g of sodium sulfide and 60 mg of elemental sulfur were dissolved in 10 mL of deionized water and treated in an oven at 80 °C overnight to obtain a yellow NaPS solution. Then, 10 mL of Ti_3_C_2_T_*x*_ suspension (6 mg mL^−1^) and 10 mL of NaPS solution were mixed and stirred well for 10 min. Afterward, formic acid was added dropwise to the above solution under stirring until the pH was close to neutral. The mixed solution was then centrifuged and washed twice with deionized water to remove impurities and the products were freeze-dried for further assembly. In short, the pure Ti_3_C_2_T_*x*_ mentioned in the assembly of Ti_3_C_2_T_*x*_/RGO hydrogel was replaced with Ti_3_C_2_T_*x*_@S to prepare MGA@S. And SMGA was obtained through heat-treatment in a tube furnace at 400 °C under N_2_ for 4 h.

### Characterizations

#### Structure Characterizations

The structures, morphologies, and elemental mappings of MGA, SMGA were investigated by a transmission electron microscope (TEM, Titan G260-300) at an accelerating voltage of 200 kV, a scanning electron microscope (SEM, Hitachi S4800), X-ray photoelectron spectrometer (XPS, AXIS SUPRA), X-ray diffraction instrument (XRD, Rigaku MiniFlex) using Ni-filtered Cu Kα radiation at a scan rate of 10° min^−1^, a Raman spectrometer (invia-reflex with 532 nm wavelength laser). The Zeta potentials of Ti_3_C_2_T_*x*_, GO, Ti_3_C_2_T_*x*_/GO, Ti_3_C_2_T_*x*_/GO&APTES solution were tested using a zeta potential analyzer (Zetasizer Nano ZSP). The specific surface areas were tested using a Micromeritics ASAP2460 based on the Brunauer–Emmett–Teller (BET) method.

#### Electrochemical Measurements

MGA and SMGA were fabricated as freestanding electrodes by slicing and pressing using a micro tableting machine (KJ-GROUP, YLJ-20). For sodium-ion cells, the electrochemical measurements were performed in CR2032 type coin half-cells, which were assembled in the argon-filled glove box with oxygen and moisture below 0.01 ppm (Mikrouna). Na foil was used as the counter electrode, glass fiber (Whatman GF/A) was used as the separator, and 1.0 M NaClO_4_ dissolved in a mixture of EC and DEC (1:1 v/v) with 5 wt% fluorinated ethylene carbonate (FEC) as the electrolyte. The CV and EIS tests were carried out on the CHI660E electrochemical workstation. Galvanostatic charge/discharge measurements were performed in a potential range of 0.01 and 3.0 V (vs. Na^+^/Na) at different current densities using a battery test system (Neware). For the sodium-ion hybrid capacitor, SMGA (about 0.6 mg) and activated carbon (YP50F) (2.4 mg) were selected as anode and cathode electrodes, respectively. And both electrodes were pre-activated in half-cells by pairing with pure Na foil. To increase the stability of the electrodes and avoid the impact of side reactions, the anode was cycled from 0.01 to 3 V for 10 times and stopped at 0.8 V, while the cathode was cycled 10 times from 2.0 to 4.0 V and stopped at 2.0 V. Specifically, the multiple cycles contribute to the formation of a stable SEI of the anode and alleviate the effects of irreversible reactions of the anode and cathode. A suitable cut-off voltage will ensure sufficient sodium ions pre-embedded the electrode. The potential window of the hybrid capacitor was set to be 0.01–4.0 V.

## Results and Discussion

**Fig. 1:**
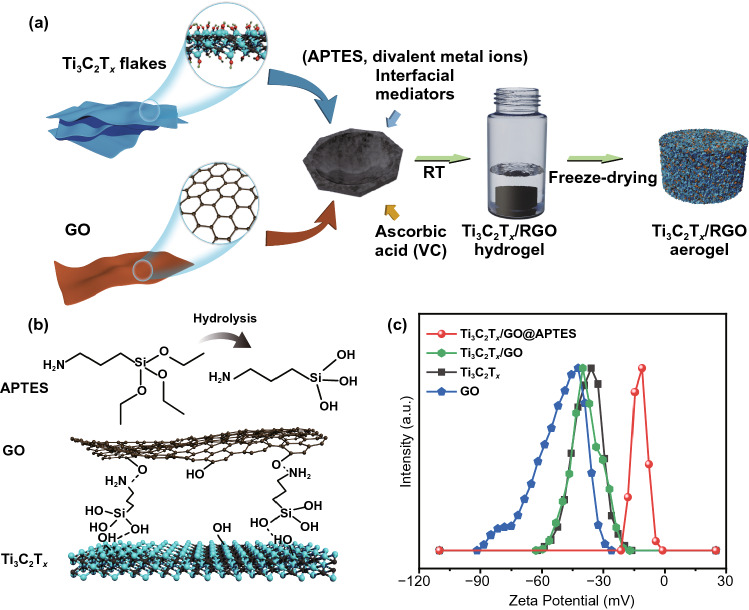
**a** Schematic illustration of the synthetic process of Ti_3_C_2_T_*x*_/RGO aerogels. **b** Diagram of the cross-linking of Ti_3_C_2_T_*x*_ and RGO nanosheets by the hydrolyzed APTES. **c** Zeta potentials of Ti_3_C_2_T_*x*_, GO, Ti_3_C_2_T_*x*_/GO, and Ti_3_C_2_T_*x*_/GO with the addition of APTES

The synthesis process of Ti_3_C_2_T_*x*_/RGO is schematically depicted in Fig. 1a. Typically, delaminated Ti_3_C_2_T_*x*_ suspension has been evenly mixed with the GO colloidal solution (10 mg ml^−1^) in a mortar. Interfacial mediators have been added to the mixture followed by continuous stirring. Then, ascorbic acid (VC) solution has been added to the above mixture for the reduction gelation of GO. Immediately afterward, the above slurry has been transferred to a glass vial. After 72 h at 25 °C, a uniform Ti_3_C_2_T_*x*_/RGO hydrogel has formed automatically and Ti_3_C_2_T_*x*_/RGO monolith has been obtained after washing and freeze-drying at -40 °C [47]. For amino-propyltriethoxysilane (APTES) as the interfacial mediator, it undergoes hydrolysis in the presence of water and the exposed silyl and cationic amino groups will interact with hydroxyl groups on the surfaces of Ti_3_C_2_T_*x*_ and GO by hydrogen bonding to form a cross-linking structure (Fig. 1b). The cross-linking effect of APTES on bridging Ti_3_C_2_T_*x*_ and GO is verified by Zeta potential analysis (Fig. 1c). Both Ti_3_C_2_T_*x*_ and GO are negatively charged, forming stable colloids in water (Fig. S1a, b). As a result, the Ti_3_C_2_T_*x*_/GO mixed colloid is stable and no aggregation is found due to the electrostatic repulsion between Ti_3_C_2_T_*x*_ and GO (Fig. S1c). After adding APTES, the Zeta potential of Ti_3_C_2_T_*x*_/GO shifts positively from − 40 to − 11 mV. This suggests that the introduction of APTES reduces the electrostatic repulsion between Ti_3_C_2_T_*x*_ and GO effectively, which is of key importance to overcome the dispersing effect of Ti_3_C_2_T_*x*_ at a high Ti_3_C_2_T_*x*_/GO mass ratio for the construction of Ti_3_C_2_T_*x*_/RGO composite aerogels. As a demonstration, when excess APTES has been added to the Ti_3_C_2_T_*x*_/GO mixed colloid directly, the flocculent cross-linked aggregation has quickly formed in the solution (Fig. S1d), which intuitively shows the cross-linking effect of APTES.

**Fig. 2:**
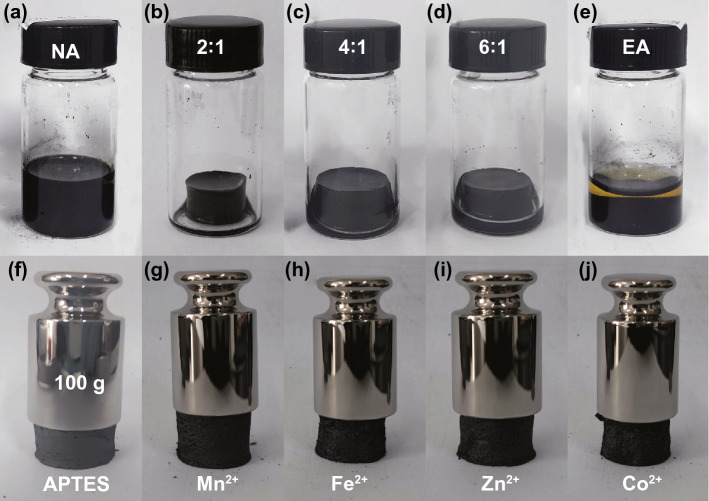
Room temperature assembly of Ti_3_C_2_T_*x*_. **a** Ti_3_C_2_T_*x*_/RGO mixture without adding interfacial mediators. **b**–**d** Ti_3_C_2_T_*x*_/RGO hydrogels obtained from different Ti_3_C_2_T_*x*_/GO ratios from 2:1 to 6:1. **e** Ti_3_C_2_T_*x*_/RGO with excessive APTES addition. **f**–**j** Photos of Ti_3_C_2_T_*x*_/RGO aerogels prepared from a Ti_3_C_2_T_*x*_/GO ratio of 6:1 by using different interfacial mediators bearing 100 g weight

For the control experiment without any interfacial mediators, no monolithic assembly has been obtained when the Ti_3_C_2_T_*x*_/GO mass ratio is 6:1 with no APTES (NA) (Fig. 2a). In contrast, the introduction of APTES leads to the successful formation of well-defined hydrogels with Ti_3_C_2_T_*x*_/GO ratios ranging from 2:1 to as high as 6:1 (Fig. 2b–d). As such, it unravels the essential role of APTES in mediating the interfacial interactions between Ti_3_C_2_T_*x*_ and GO in the construction of monoliths with a high Ti_3_C_2_T_*x*_ content by the GO-assisted assembly at room temperature. Notably, excessive APTES (EA) has caused fast aggregation of Ti_3_C_2_T_*x*_/GO mixed colloid, resulting in the generation of sediment instead of hydrogel, as shown in Fig. 2e. Appropriate amount of positively charged APTES will crosslink the negatively charged Ti_3_C_2_T_*x*_ nanosheets and GO nanosheets. In this case, the mixed solution can remain relatively stable and form a uniform 3D structure. However, excessive APTES hydrolysates to cross-link with Ti_3_C_2_T_*x*_ nanosheets, which will destroy the electrostatic equilibrium of colloid solution and limit the formation of the uniform porous gel structure. The aerogel prepared from a nominal Ti_3_C_2_T_*x*_/GO mass ratio of 6:1 is denoted as MGA (MXene/RGO aerogel), which can support 100 g weight without significant deformation (Fig. 2f). This suggests that a small ratio of GO can form a 3D continuous network with the assistance of the interfacial mediator of APTES for the encapsulation of Ti_3_C_2_T_*x*_ nanosheets to form a robust monolith with promising mechanical strength. Based on the weights of MGA (62 mg) and bare RGO aerogel (8 mg) prepared in a similar method without Ti_3_C_2_T_*x*_, it is estimated that the ratio in the resulting MGA is around 87 wt%. Further experiment shows that it is feasible to scale up the method presented here to construct a larger MGA with a diameter of 7.8 cm (Fig. S2). Inspired by the role of APTES in screening the electrostatic repulsion between Ti_3_C_2_T_*x*_ and GO, different cationic metal ions have been selected as similar interfacial mediators for the construction of Ti_3_C_2_T_*x*_-based monoliths under the same condition. As shown in Fig. 2g–j, various metal ions (Mn^2+^, Fe^2+^, Zn^2+^, and Co^2+^) have also realized the successful assembly of monoliths at a Ti_3_C_2_T_*x*_/GO mass ratio of 6:1 with good structural robustness. The structural robustness is highly desirable for practical applications where integrated Ti_3_C_2_T_*x*_-based functional materials are required. Noteworthily, Yang and coworkers have shown that divalent metal ions lead to the 3D gelation of Ti_3_C_2_T_*x*_ colloid [29]. Divalent metal ions are advantageous in terms of cross-linking while avoiding the oxidation of Ti_3_C_2_T_*x*_ than monovalent and trivalent metal ions. Unfortunately, it is found that the aerogels obtained by cross-linking Ti_3_C_2_T_*x*_ directly with metal ions in the absence of GO show poor mechanical strength compared to the counterparts with GO, which have been readily broken down into powders under pressing (Fig. S3). More encouragingly, the room-temperature GO-assisted assembly of Ti_3_C_2_T_*x*_ MXene modulated by interfacial mediators is feasible for further modifications by incorporating other functional nanomaterials with Ti_3_C_2_T_*x*_ as the starting material. As a paradigm, we have used sulfur nanoparticles loaded Ti_3_C_2_T_*x*_ nanosheets (S/Ti_3_C_2_T_*x*_) in a similar way to produce S@MGA while maintaining a same Ti_3_C_2_T_*x*_/GO ratio of 6:1 to that of MGA, and sulfur modified MGA (SMGA) was prepared after eliminating sulfur nanoparticles by heat-treatment at 400 °C (Fig. S4). The obtained SMGA exhibits excellent mechanical integrity and remains in hydrophobic state in continuous ultrasonic treatment for 150 min (Fig. S5), demonstrating the good structural stability.

**Fig. 3:**
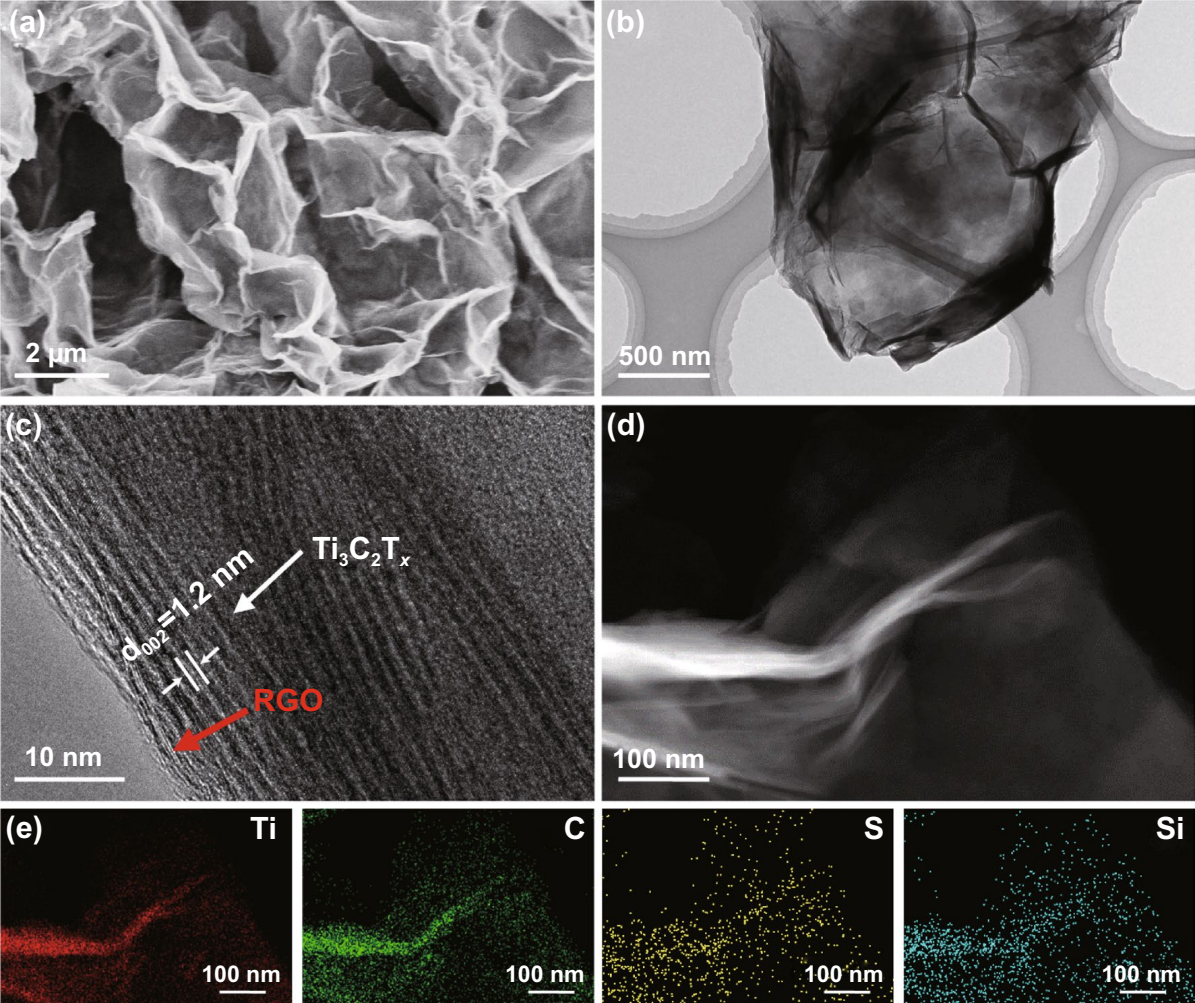
**a** SEM image, **b**–**c** TEM images, d high-angle annular dark-field image with e the corresponding EDS mapping of SMGA

The microstructures have been characterized by scanning electron microscopy (SEM). As shown in Fig. S6, MGA has a typical 3D structure formed by interconnected nanosheets, which prevents aggregation of the 2D nano-building blocks, thereby promoting the surface accessibility for practical applications. Similarly, SMGA has a 3D interconnected porous structure identical to MGA, showing that the developed protocol for the 3D assembly of Ti_3_C_2_T_*x*_ is viable for the further sulfur modification process (Fig. 3a). The morphology and interfacial interactions between Ti_3_C_2_T_*x*_ and RGO in SMGA have been investigated by TEM. As shown in Fig. 3b, a visible porous structure is observed, which is consistent with the SEM observations. Specially, no particulate TiO_2_ is present, indicating that the oxidation of Ti_3_C_2_T_*x*_ is effectively suppressed. In the high-resolution TEM (HRTEM) image (Fig. 3c), Ti_3_C_2_T_*x*_ nanosheets could be easily distinguished from RGO nanosheets through the layer thickness because RGO consists of a single C layer while three Ti layers are interspersed alternately with two C layers for Ti_3_C_2_T_*x*_. And the intimate interfacial contact between Ti_3_C_2_T_*x*_ and RGO nanosheets are observed, which confirms the effective cross-linking between Ti_3_C_2_T_*x*_ and GO. Additionally, the elemental mapping analysis shows the homogeneous distribution of Ti, C, S, and Si (Fig. 3d, e). Among them, the presence of Si confirms the essential role of APTES in bridging Ti_3_C_2_T_*x*_ and RGO. Since no obvious sulfur nanoparticles is observed in the TEM image in Fig. 3b, it is believed that S exists primarily as the lattice dopant in the resulting SMGA, which will be further elaborated in the following discussions. And there is no TiO_2_ nanoparticles on the smooth surface of the obtained sample in TEM image, further indicating that the Ti_3_C_2_T_*x*_ has not been oxidized significantly.

**Fig. 4:**
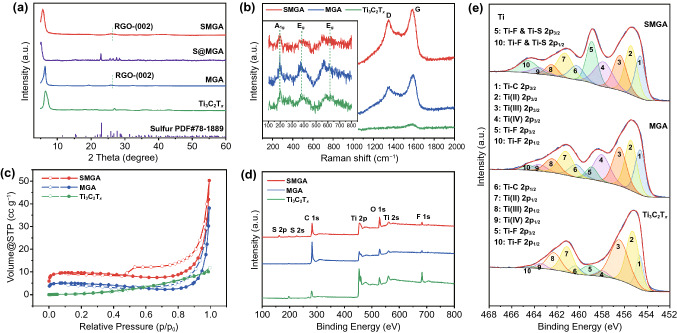
**a** XRD patterns of the Ti_3_C_2_T_*x*_, MGA, MGA@S, and SMGA. **b** Raman spectra of Ti_3_C_2_T_*x*_, MGA, and SMGA. **c** N_2_ adsorption–desorption isotherms of Ti_3_C_2_T_*x*_, MGA and SMGA. **d** XPS survey spectra of Ti_3_C_2_T_*x*_, MGA, and SMGA. **e** Ti 2p spectra of Ti_3_C_2_T_*x*_, MGA, and SMGA

Figure 4a shows the XRD patterns of the samples. The prominent peaks at around 6° corresponding to the (002) crystal plane of Ti_3_C_2_T_*x*_ are observed in all samples. Compared with bare Ti_3_C_2_T_*x*_ film prepared by vacuum filtration of Ti_3_C_2_T_*x*_ colloid, the (002) diffraction peak for MGA shifts from 6.5° to 6.3°, which indicates the effective suppression of the face-to-face restacking due to the 3D assembly. In addition, a diffraction appears to peak at 25° for MGA, corresponding to the (002) crystal plane of RGO. For S@MGA, the typical diffraction peaks of S are discernable and the (002) diffraction peak of Ti_3_C_2_T_*x*_ further downshifts to 5.1°, which suggests the introduction of sulfur nanoparticles is effective to alleviate restacking of Ti_3_C_2_T_*x*_ nanosheets. Compared to S@MGA, the diffraction peaks of S nanoparticles disappear for SMGA, which is in line with the TEM results. More importantly, the Ti_3_C_2_T_*x*_ (002) peak for the SMGA shifts to smaller angles than that of MGA, which indicates the enlarged interlayer distance after sulfur modification. There is no obvious characteristic peak of anatase TiO_2_ for both MGA and SMGA samples, which show that the Ti_3_C_2_T_*x*_ in the aerogel has not been significantly oxidized. The compositions of the as-prepared specimens have been further investigated by Raman spectroscopy. As shown in Fig. 4b, MGA and SMGA present obvious D and G bands of RGO at 1349 and 1589 cm^−1^, respectively [48]. Besides, the MGA and SMGA monoliths show the typical modes identical to that of Ti_3_C_2_T_*x*_ film before 800 cm^−1^. Specifically, the mode at 198 cm^−1^ is the A_1g_ symmetry out-of-plane vibrations of Ti atoms, while the modes at 377 and 623 cm^−1^ are the E_g_ group vibrations, including in-plane (shear) modes of Ti, C, and surface functional group atoms [49, 50]. Noteworthily, no Raman mode of the Ti_3_C_2_T_*x*_ oxidation degradation product of TiO_2_ is detected, which agrees with the TEM and XRD results, consolidating the stability of Ti_3_C_2_T_*x*_ during the assembling process. This is pivotal to deliver the attractive physicochemical properties of Ti_3_C_2_T_*x*_ nanosheets in 3D monolithic form for target applications. The sulfur nanoparticles can reduce the restacking of Ti_3_C_2_T_*x*_ nanosheets to increase the exposed surface area, which has been further confirmed by the N_2_ adsorption/desorption measurements (Fig. 4c). Both samples exhibit typical type-IV behavior with a distinct hysteresis loop of type H3. The specific surface area of SMGA (29.2 m^2^ g^−1^) is higher than that of pure Ti_3_C_2_T_*x*_ (6.7 m^2^ g^−1^) and MGA (14.3 m^2^ g^−1^), indicative of the improved surface accessibility of Ti_3_C_2_T_*x*_ by the 3D assembly and sulfur modification.

XPS has been used to investigate the surface chemistry of the assembled aerogels. As shown in Fig. 4d, the predominant signals of C 1 s, Ti 2p, Ti 2 s, O 1 s, and F 1 s are found in the survey XPS spectra of the Ti_3_C_2_T_*x*_, MGA, and SMGA. Additional peaks at around 164 and 227 eV are observed for SMGA, corresponding to S 2 s and S 2p peaks, respectively. Thermogravimetric analysis (TGA) shows that SMGA has an almost identical mass loss behavior to MGA during heat-treatment to 800 °C (Fig. S7). Thus, it can be inferred that there is almost no residual elemental sulfur in SMGA, which agrees well with the TEM and XRD results as aforementioned. As shown in the S 2p XPS spectrum (Fig. S8), the existence of Ti-S bond indicates that sulfur bonds with Ti_3_C_2_T_*x*_. Furthermore, the two peaks at 163.5 and 164.5 eV corresponding to the terminal and the bridging S–S bonds can be identified as a sulfur chain fixed to the surface of Ti atoms. This suggests that the S element modifies the surface of Ti_3_C_2_T_*x*_ by substituting the functional group on its surface [51]. For Ti 2p XPS spectra of bare Ti_3_C_2_T_*x*_ and MGA, the peaks at 459.0 and 464.5 eV are assigned to Ti 2p_3/2_ and Ti 2p_1/2_ of Ti-F, respectively (Fig. 4e). Obviously, the relative intensities of this pair of peaks increase for SMGA, which could be ascribed to the emergence of Ti-S bonds. The combined characterization results show that sulfur modification not only increases the surface accessibility of the monolith but also leads to the sulfur doping of Ti_3_C_2_T_*x*_. Furthermore, Si XPS peak originating from APTES is observed for MGA and SMGA (Fig. S9), which is in accordance with the EDS mapping results.

**Fig. 5:**
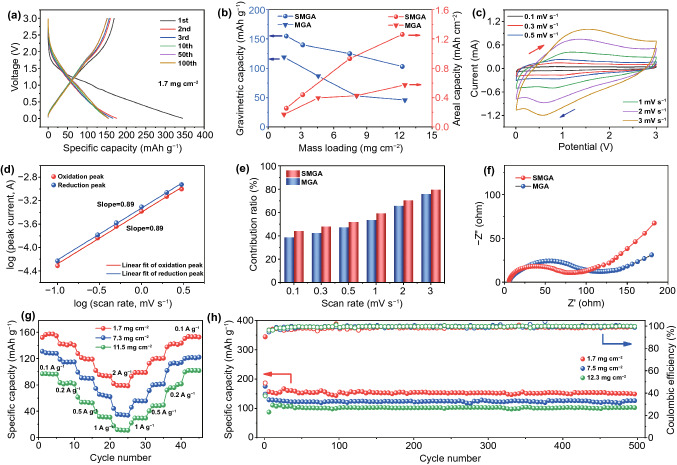
Half-cell tests of the freestanding SMGA and MGA electrodes for electrochemical sodium-ion storage. **a** Galvanostatic charge–discharge profiles of SMGA at 100 mA g^−1^. **b** Gravimetric and areal capacities of SMGA and MGA with different areal densities at 100 mA g^−1^. **c** CV curves of the SMGA electrode at different scan rates from 0.1 to 3 mV s^−1^. **d** Relationship between the peak current and scan rate for the SMGA electrode. **e** Capacitance contributions of SMGA and MGA at different scan rates. **f** Nyquist plots of SMGA and MGA in Na-ion half-cell. **g** Rate performances of the SMGA electrodes with different mass loadings at rates ranging from 0.1 to 5 A g^−1^. **h** Cycling performances and Coulombic efficiencies of the SMGA electrodes with different mass loadings at a current density of 0.1 A g^−1^

To verify the electrochemical advantages of the obtained aerogel and evaluate the effect of the sulfur modification, we have tested the electrochemical properties of SMGA and MGA as the freestanding electrode for sodium-ion storage. Quasi-linear galvanostatic charge–discharge curves without apparent charge/discharge plateaus are observed for MGA (Fig. S10) and SMGA (Fig. 5a). Specifically, the MGA electrode with a mass loading of 1.5 mg cm^−2^ delivers a discharge capacity of 257 mAh g^−1^ with a charge capacity of 112 mAh g^−1^ in the first cycle at 100 mA g^−1^, where the capacity loss can be ascribed to the formation of solid electrolyte interface (SEI) and the side reaction of the electrolyte [52]. Notably, SMGA with a similar mass loading of 1.7 mg cm^−2^ delivers a higher reversible capacity of 155 mAh g^−1^ than MGA in the first cycle at 100 mA g^−1^, demonstrating that the sulfur modification is effective to boost the electrochemical sodium-ion storage performance. Specifically, as shown in Fig. S11, the SMGA maintains its capacity advantage and rate performance over MGA in the long-term cycling. And both electrodes show better electrochemical sodium storage performance than pure Ti_3_C_2_T_*x*_. As shown in Fig. 5b, the capacity gap between SMGA and MGA gradually increases as the mass loading goes up. Compared with other freestanding MXene-based electrodes for sodium-ion storage, SMGA shows excellent areal capacity [53]. Notably, even with a high mass loading of 12.3 mg cm^−2^, SMGA retains a reversible gravimetric capacity of 102 mAh g^−1^, corresponding to an areal capacity of 1.26 mAh cm^−2^. To investigate the effect of sulfur modification on the enhanced electrochemical performances, the electrochemical charge storage kinetics of the SMGA and MGA electrode have been investigated by cyclic voltammetry (CV) at scan rates from 0.1 to 3 mV s^−1^ (Figs. 5c and S12a). A pair of broad cathodic/anodic peaks located at around 0.5/1.4 V are observed, corresponding to the electrochemical reaction between Na^+^ and Ti_3_C_2_T_*x*_. Figures 5d and S12b show the relationships between the anodic/cathodic peak current (*i*) and the scan rate (*v*) of the SMGA and MGA, respectively. And the slopes of the linearly fitted plots of log(*v*) − log(*i*) for the cathodic/anodic peaks are 0.84/0.83 for MGA (Fig. S12b) and 0.89/0.89 for SMGA (Fig. 5d), which suggests that the charge storage process in the is predominantly controlled by a fast capacitive behavior [54]. The contributions of surface-driven capacitive reactions at different scan rates (*k*_1_ν) can be evaluated based on the formula of *i* = *k*_1_ν + *k*_2_ν^1/2^ [7]. As summarized in Fig. 5e, SMGA shows a higher capacitive contribution of 44.4% than MGA (38.8%) at 0.1 mV s^−1^. The proportions of the capacitive contribution gradually increase at higher scan rates for both MGA and SMGA, and the value for SMGA remains higher than that of MGA. The Nyquist plots for the SMGA and MGA electrodes are compared in Fig. 5f. A steeper sloping line in the low-frequency region for SMGA than that of MGA is observed, which suggests a better electrolyte ion diffusion in the SMGA electrode due to the improved surface accessibility as evidenced by the previous BET surface area results. It is also noted that the charge transfer impedance (*R*_ct_) of SMGA decreases to 50.9 Ω as compared to the counterpart of MGA (59.8 Ω), evidencing the enhanced charge transfer kinetics in SMGA. Linear fit of the Warburg impedance of SMGA and MGA electrodes correlates to different Na^+^ diffusion efficiency. The decreased slope of the Z′-ω^−1/2^ curve for the SMGA electrode compared to that of MGA demonstrates improved Na^+^ diffusion efficiency (Fig. S13) [55]. As shown in Fig. S14, it is measured that the electrical conductivity of SMGA is 5190 S cm^−1^, which is higher than that of MGA (4210 S cm^−1^). In addition, a previous theoretical investigation shows that the surface sulfur doping of Ti_3_C_2_T_*x*_ reduced the Na^+^ diffusion barrier [41]. Consequently, these factors cooperatively contribute to boosting the charge transfer kinetics in SMGA. Based on the investigations, the improved electrochemical performances of SMGA compared to MGA for sodium-ion storage can be attributed to the sulfur modification effect on: (i) improving the surface accessibility of SMGA compared to MGA, and (ii) enhancing the charge transfer kinetics by increasing the electrical conductivity and reducing the Na^+^ diffusion barrier. The rate capability of the SMGA electrode with incremental areal density at different current densities (from 0.1 to 2 A g^−1^) is shown in Fig. 5g. At a high current density of 2 A g^−1^, the SMGA electrode with a mass loading of 1.7 mg cm^−2^ delivers a capacity of 80 mAh g^−1^, while a thicker electrode with a mass loading of 11.5 mg cm^−2^ achieves a capacity of 11 mAh g^−1^. Figure 5h displays the long-cycling profile of the SMGA electrode at the current density of 0.1 A g^−1^. There is almost no capacity decay for all electrodes from the 10th to 500th cycle and discharge capacities of 155, 122, and 102 mAh g^−1^ are obtained after 500 cycles for the SMGA electrode with a mass loading of 1.7, 7.5, and 12.3 mg cm^−2^, respectively.

**Fig. 6:**
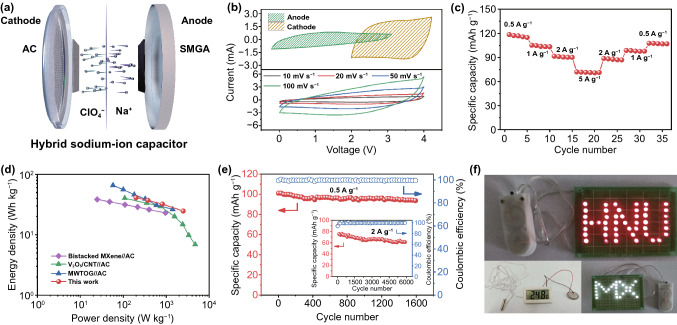
**a** Schematics of the assembled AC//SMGA hybrid sodium-ion capacitor (SIC). **b** CV curves of SMGA and AC in a Na-ion half-cell at a scan rate of 10 mV s^−1^ (top part) and AC//SMGA SIC at different scan rates (bottom part). **c** Rate performances of the AC//SMGA SIC at rates ranging from 0.5 to 5 A g^−1^. **d** Ragone plots of the AC//SMGA SIC compared to previously reported SICs: Bistacked MXene//AC [57], V_2_O_5_/CNT//AC [58], and TiO_2_ mesocage-graphene nanocomposite (MWTOG)//AC [59]. **e** Long-term cycling performances and Coulombic efficiencies of the AC//SMGA SIC at current densities of 0.5 A g^−1^ and 2 A g^−1^ (inset). **f** Digital photos of LED arrays and electronic thermometer powered by the assembled AC//SMGA SIC

To further confirm the practical feasibility of the 3D porous composite aerogel for electrochemical energy storage, hybrid sodium-ion capacitors (SIC) are assembled by employing SMGA as the anode and commercial activated carbon (AC) as the cathode (Fig. 6a). As shown in Fig. 6b (top part), the AC cathode has been tested in the potential window of 2–4 V (vs Na^+^/Na), which shows a CV curve of a typical rectangular shape corresponding to a reversible adsorption–desorption charge storage mechanism of anionic ClO_4_^−^ on the surface of AC. And the spindle-shaped anode profile tested at 0.01–3 V represents the electrochemical process of Na^+^ in the SMGA electrode. The charge balance between the cathode and anode is controlled based on the equation of m_+_C_+_  = m_–_C_–_, where m_+_ and m_–_ are the mass of the activated material, C_+_ and C_-_ are the specific capacity of anode and cathode, respectively [56]. Consequently, the active material mass ratio of the cathode/anode (m_+_/m_–_) is determined to be about 4:1 based on the specific capacities of AC and SMGA (Fig. S14). CV curves of the assembled AC//SMGA SIC at scan rates of 10–100 mV s^−1^ are shown in Fig. 6b (bottom part). The slightly distorted quasi-rectangular CV curves imply the mixed Faradaic and non-Faradaic charge-storage process. The rate capability of the AC//SMGA SIC at different current densities (from 0.5 to 5 A g^−1^) is shown in Fig. 6c. At a high current density of 5 A g^−1^, the SMGA electrode still delivers a capacity of 70 mAh g^−1^, which is a vital performance indicator for SIC devices requiring fast charge and discharge. The energy and power densities of the SIC have been calculated based on the total weight of AC and SMGA. As shown in Fig. 6d, compared with the SICs reported previously [57–59], the SIC assembled with SMGA shows competing energy densities with outstanding power densities. Typical device energy densities of 41 and 25 Wh kg^−1^ have been achieved at the power densities of 197 and 2473 W kg^−1^, respectively. Stable cycling performance is also demonstrated for the AC//SMGA SIC (Fig. 6e). Specifically, the AC//SMGA SIC displays a reversible capacity of 93.7 mAh g^−1^ (calculated based on the mass of the anode) after 1600 cycles with a high Coulombic efficiency of above 99.5% at a current density of 0.5 A g^−1^, corresponding to a capacity retention of 92.9%. And the assembled SIC shows a specific capacity of 60.5 mAh g^−1^ after 6000 cycles at a current density of 2 A g^−1^, which corresponds to a 67% capacity retention. For a more intuitive demonstration, the AC//SMGA SICs have been used to power various electronic devices (Fig. 6f). A few English letters composed of 47 light-emitting diodes (LEDs) can be illuminated by two AC//SMGA SICs, and a digital thermometer is successfully powered by one SIC.

## Conclusions

In summary, Ti_3_C_2_T_*x*_/RGO aerogel has been successfully prepared by GO-assisted assembly of Ti_3_C_2_T_*x*_ MXene at room temperature in the presence of interfacial mediators, including APTES, Mn^2+^, Fe^2+^, Zn^2+^, and Co^2+^. The interfacial mediators screen the electrostatic repulsion between Ti_3_C_2_T_*x*_ and GO effectively to overcome the dispersing effect of Ti_3_C_2_T_*x*_ for the construction of Ti_3_C_2_T_*x*_/RGO monolithic aerogels with a high Ti_3_C_2_T_*x*_ content of 87 wt.%. The assembled Ti_3_C_2_T_*x*_/RGO monolithic aerogel presents excellent structure stability and porous morphology without significant oxidation degradation of Ti_3_C_2_T_*x*_. On this basis, a further sulfur modification process has been introduced, which is effective to enhance the surface accessibility and charge storage kinetics for electrochemical sodium-ion storage. When fabricated as freestanding electrodes, the electrode with a practical-level mass loading of 12.3 mg cm^−2^ still delivers an areal capacity of 1.26 mAh cm^−2^ at a current density of 0.1 A g^−1^. And the promising performances in SIC have been demonstrated by pairing with activated carbon cathode. This work proves the feasibility of the GO-assisted gelation process of MXene at room temperature to produce monoliths with a high MXene content and suppressed MXene oxidation degradation, providing more possibilities for the applications of 3D MXene-based aerogels.

## Supplementary Information

Below is the link to the electronic supplementary material.

Supplementary file1 (PDF 1076 kb)
