# Supplementary material for: Room-Temperature Assembled MXene-Based Aerogels for High Mass-Loading Sodium-Ion Storage
Source: Nanomicro Lett. 2021 Dec 17;14:37. doi: 10.1007/s40820-021-00781-6 (PMC8683516; doi:10.1007/s40820-021-00781-6)
Supplement: Supplementary file 1 — Supplementary file1 (PDF 1076 kb) [file 40820_2021_781_MOESM1_ESM.pdf]

Supplementary Information for

Room-temperature Assembled MXene-based Aerogels for High Mass-Loading Sodium-Ion Storage

Fei Song[1], Jian Hu[1], Guohao Li[1], Jie Wang[1], Shuijiao Chen[2], Xiuqiang Xie[1,] *, Zhenjun Wu[2,] *, and Nan Zhang[1,] *

[1] College of Materials Science and Engineering, Hunan University, Changsha 410082, P. R. China

[2]College of Chemistry and Chemical Engineering, Hunan University, Changsha 410082, P. R. China

*Corresponding authors. E-mail: xiuqiang_xie@hnu.edu.cn (Xiuqiang Xie); nanzhang@hnu.edu.cn (Nan Zhang); wooawt@hnu.edu.cn (Zhenjun Wu)

Supplementary Figures

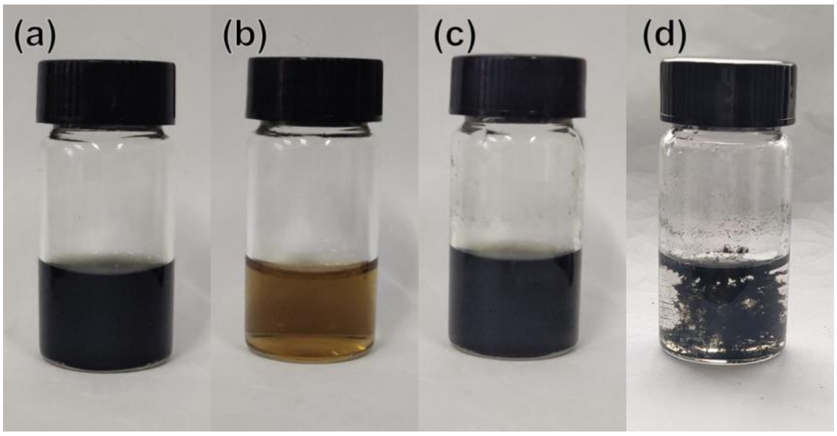

Fig. S1 Digital photos of the diluted solution of (**a**) $Ti_3C_2T_x$, (**b**) GO, (**c**) $Ti_3C_2T_x$/GO mixture and (**d**) $Ti_3C_2T_x$/GO-APTES

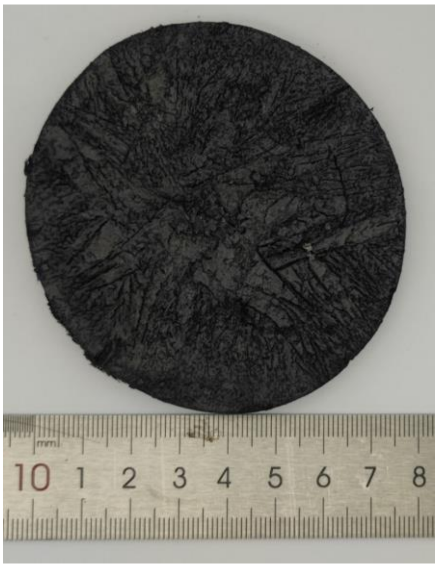

Fig. S2 Digital photo of the MGA sample with a larger volume

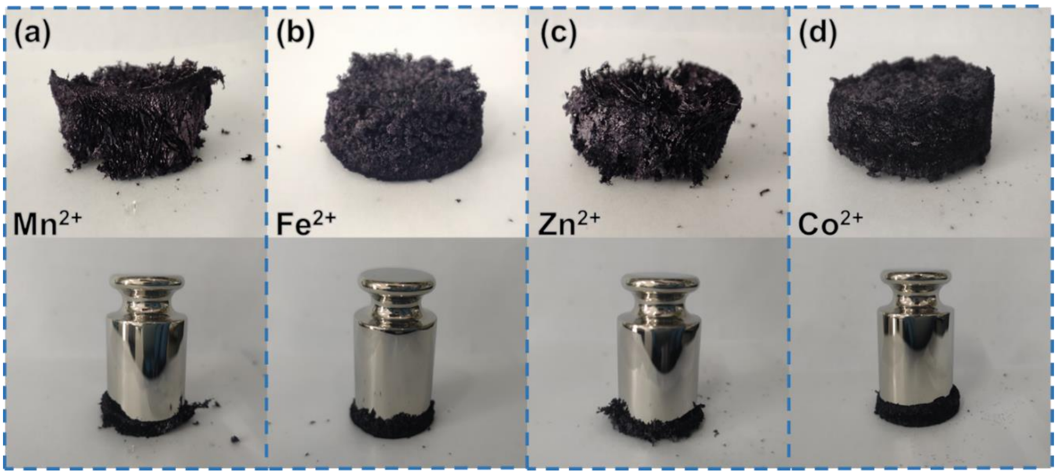

Fig. S3 $Ti_3C_2T_x$ aerogels obtained by using different metal ions (Mn^{2+}, Fe^{2+}, Zn^{2+}, and Co^{2+}) and the corresponding demonstrations bearing 100 g weight

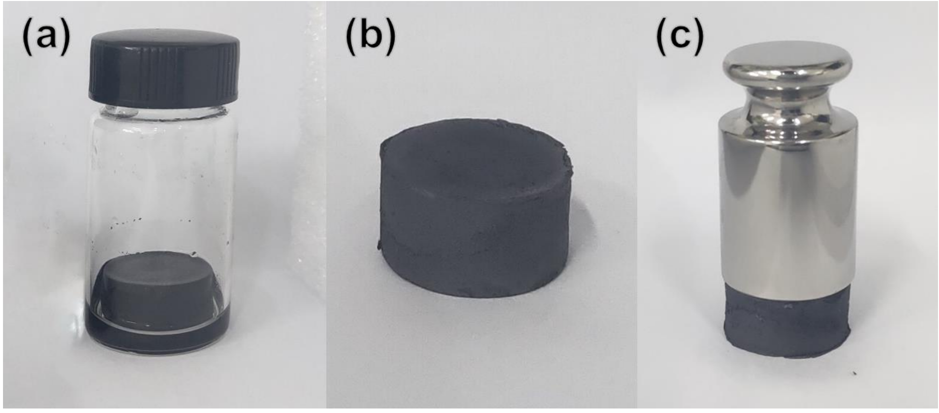

Fig. S4 Digital photos of: (**a**) MGA@S hydrogel, (**b**) SMGA, and (**c**) SMGA bearing 100 g weight

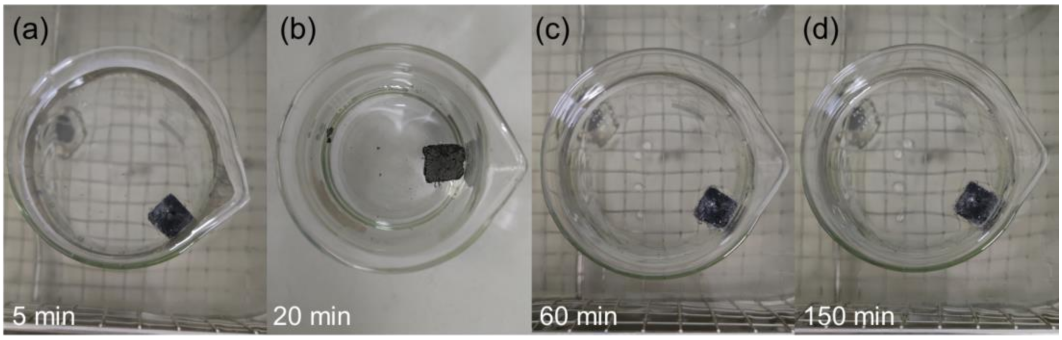

Fig. S5 Digital photos of SMGA floating on water under ultrasonication for (**a**) 5 min, (**b**) 20 min, (**c**) 60 min, and (**d**) 150 min

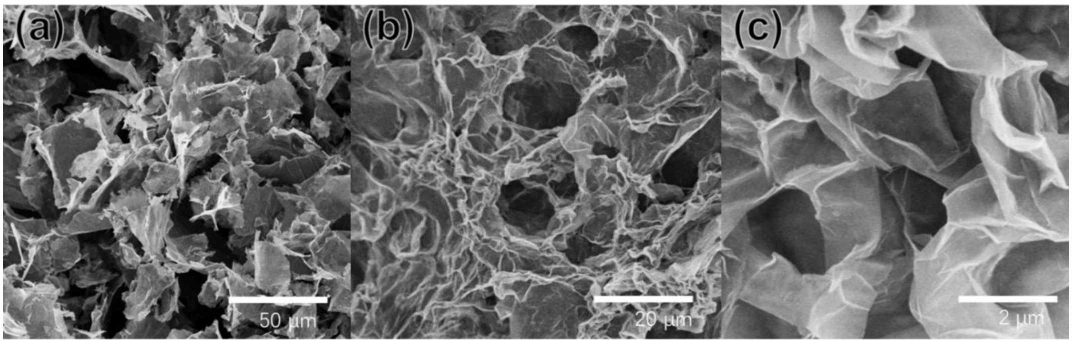

Fig. S6 SEM images of MGA at different magnifications

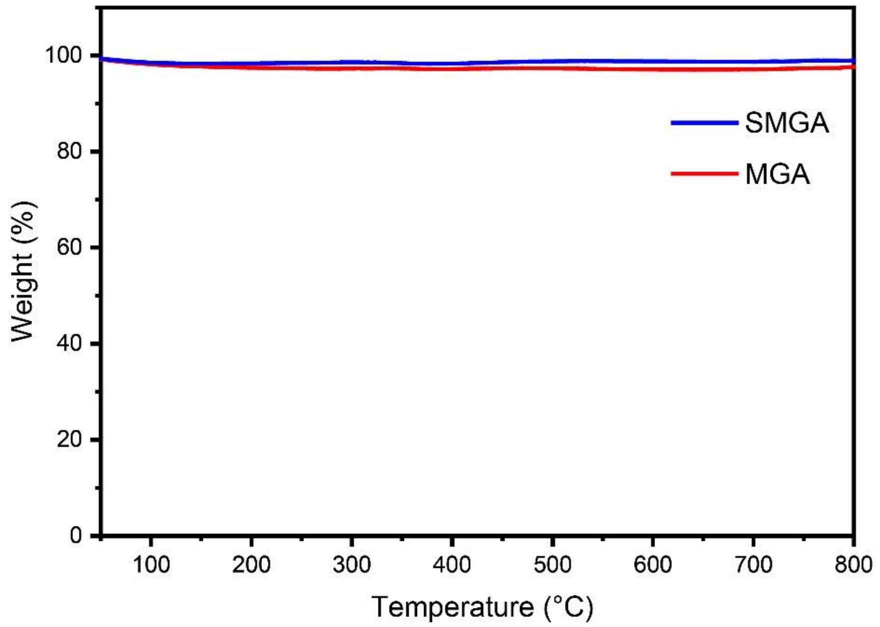

Fig. S7 TGA curves of SMGA and MGA

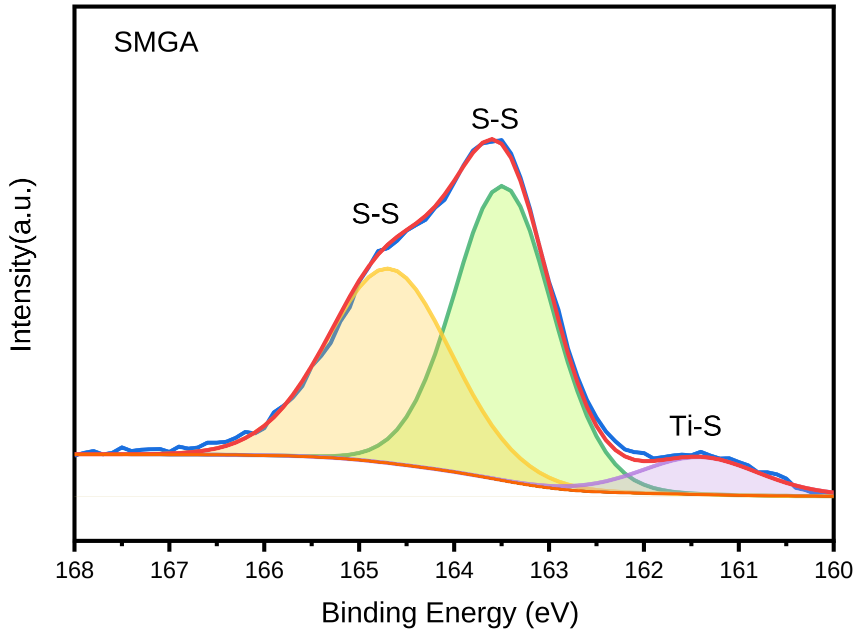

Fig. S8 S 2p XPS spectrum of SMGA

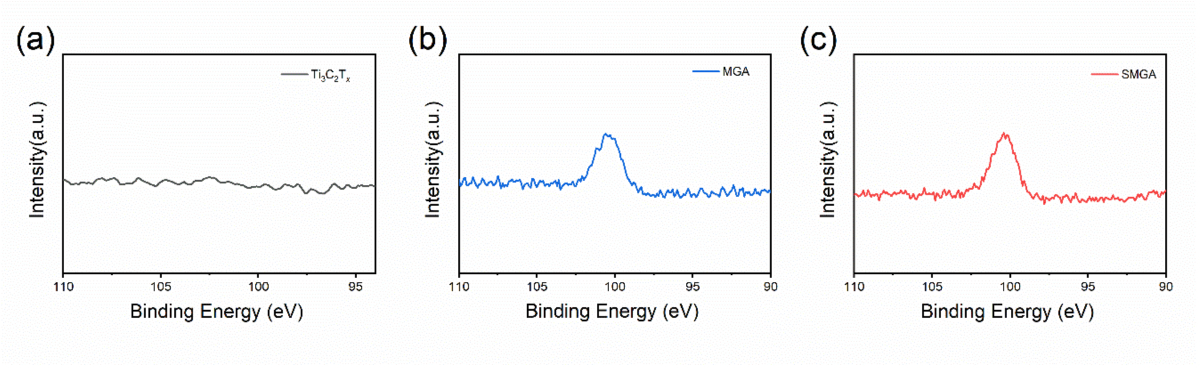

Fig. S9 Si 2p XPS spectrum of: (**a**) $Ti_3C_2T_x$, (**b**) MGA, (**c**) and SMGA

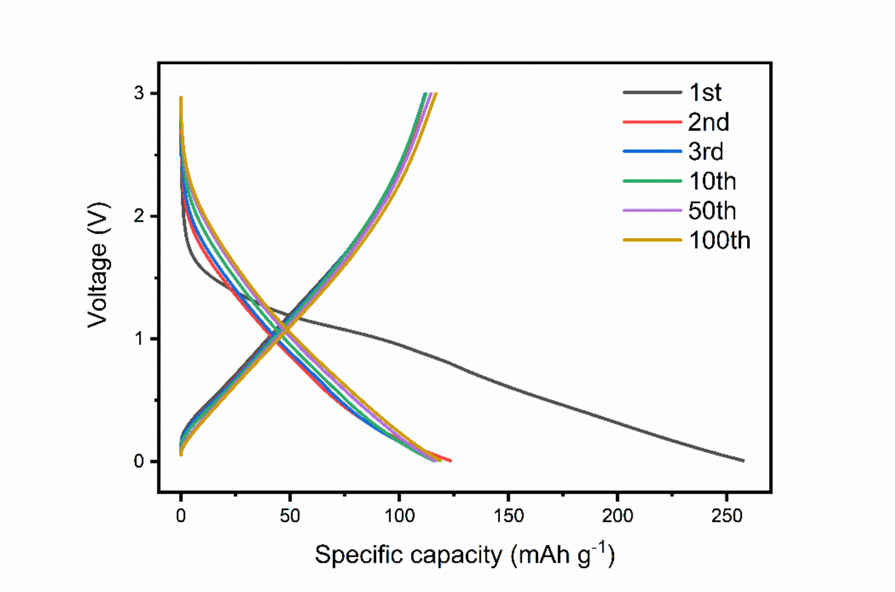

Fig. S10 Galvanostatic charge-discharge profiles of MGA (1.5 mg cm^{-2}) at 100 mA g^{-1}

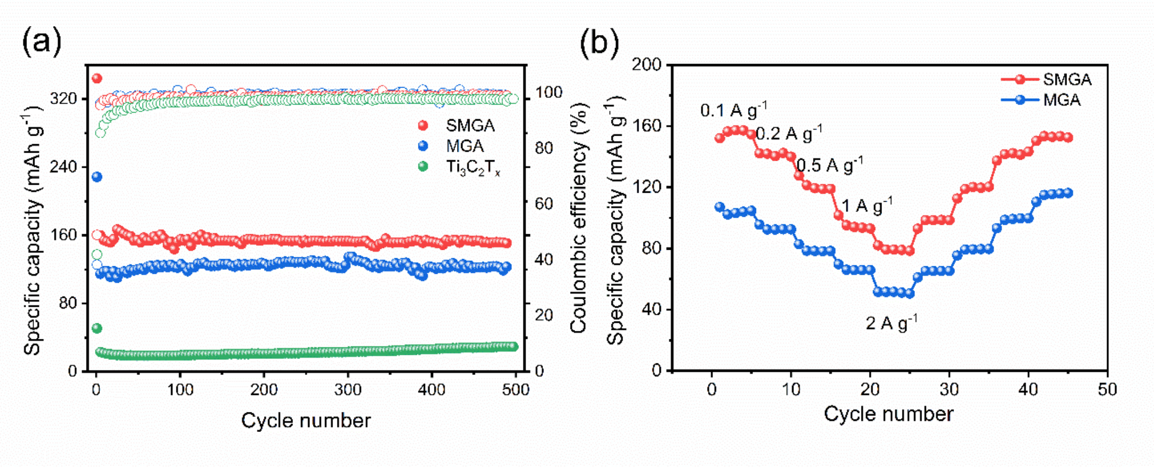

Fig. S11 (**a**) Long-term cycling performances and Coulombic efficiencies of SMGA, MGA, and pure $Ti_3C_2T_x$ at a current density of 0.1 A g^{-1}. (**b**) Rate performances of SMGA and MGA

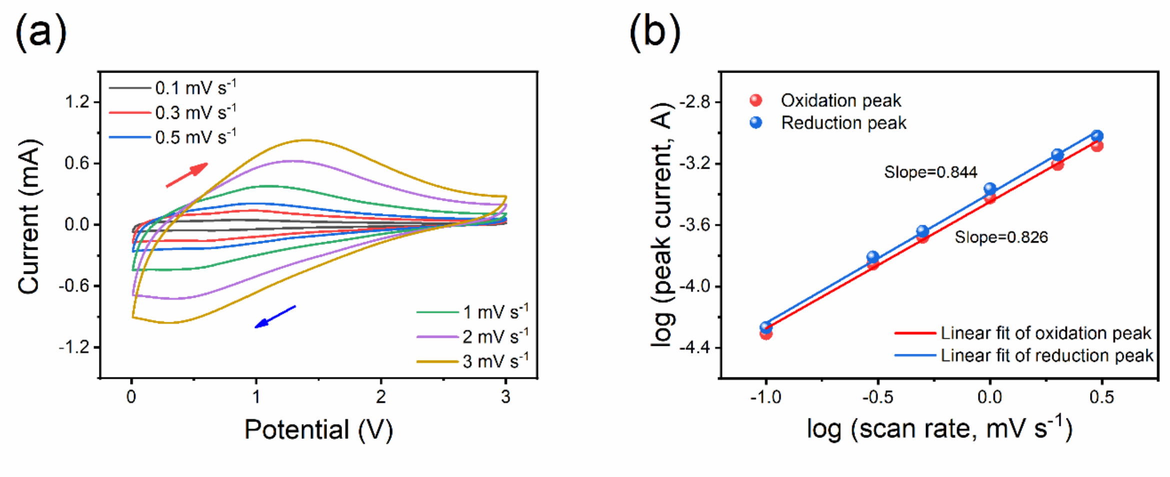

Fig. S12 (**a**) CV curves of the MGA electrode at different scan rates from 0.1 to 3 mV s^{-1}. (**b**) Relationship between the peak current and scan rate for the MGA electrode

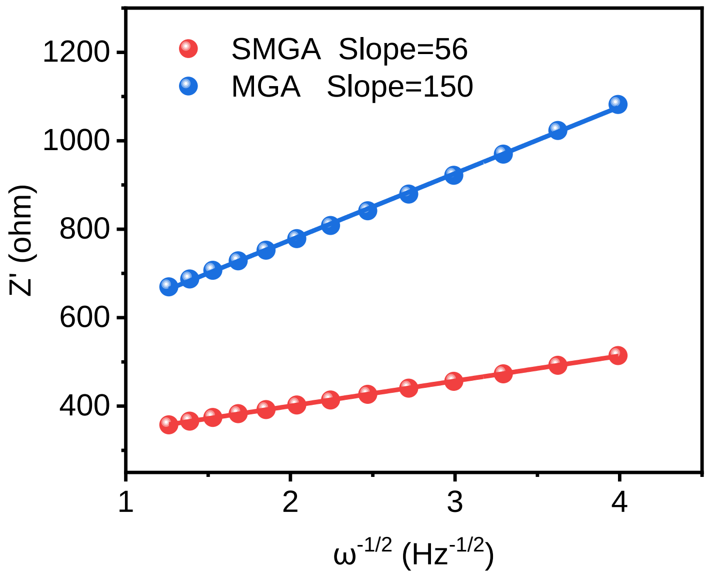

Fig. S13 Linear fit of the Warburg impedance of SMGA and MGA

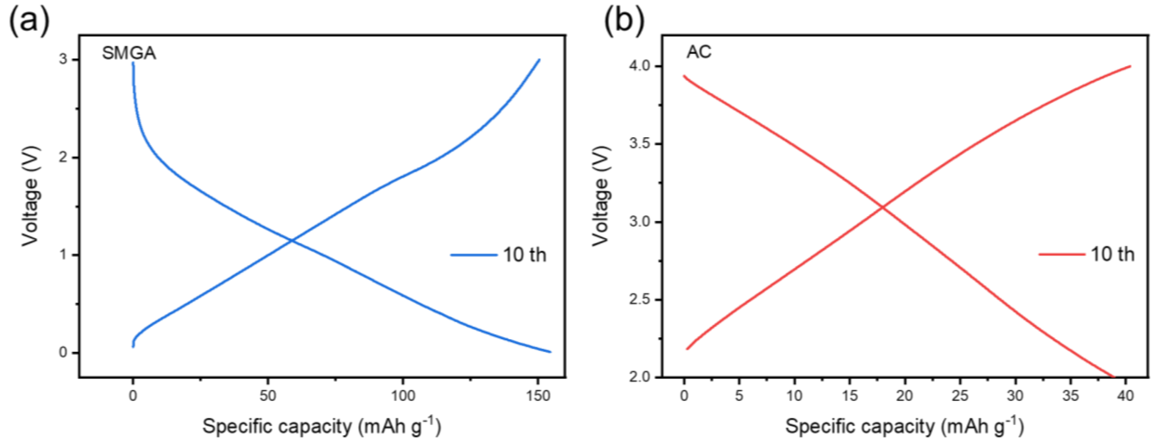

Fig. S14 Galvanostatic charge-discharge profiles of (**a**) SMGA and (**b**) AC after 10 cycles at 100 mA g^{-1}

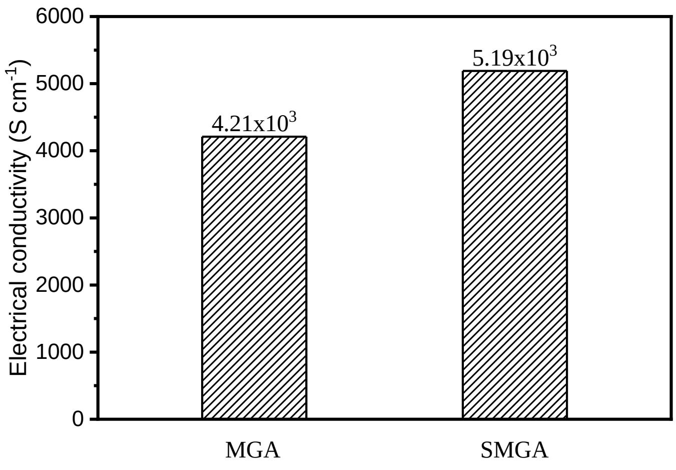

Fig. S15 The electrical conductivities of MGA and SMGA

Table S1 Comparison of the GO assisted assembly of 3D MXene recently reported

Materials	Form	Key assembly method	MXene ratio (wt%)	Reaction temperature	Refs.
$Ti_3C_2T_x$/RGO	Aerogel	Ascorbic acid reduction	15-80	65 °C	[S1]
$Ti_3C_2T_x$/RGO	Aerogel	HI reduction	30-90	80 °C	[S2]
$Ti_3C_2T_x$/RGO	Aerogel	Freeze-drying	5-20	200 °C	[S3]
$Ti_3C_2T_x$/RGO	Aerogel	Freeze-drying	6-37	60 °C	[S4]
$Ti_3C_2T_x$/RGO	Foam	Solvothermal treatment	10-25	180 °C	[S5]
$Ti_3C_2T_x$/RGO	Hydrogel	$NaHSO_3$ reduction	30-70	70 °C	[S6]
$Ti_3C_2T_x$/RGO	Hydrogel	EDA crosslinking	65-100	95 °C	[S7]
Nb_2C/RGO	Aerogel	PDDA crosslinking	50	RT	[S8]
$Ti_3C_2T_x$/RGO	Aerogel	EDA crosslinking	90	95 °C	[S9]
$Ti_3C_2T_x$/RGO	Aerogel	Dipping	15-39	120 °C	[S10]
$Ti_3C_2T_x$/RGO	Aerogel	Ascorbic acid reduction	10-30	95 °C	[S11]
Pt-$Ti_3C_2T_x$/RGO	Aerogel	K_2PtCl_4	10-90	100 °C	[S12]
$Ti_3C_2T_x$/RGO	Foam	Zn foil reduction	10-70	RT	[S13]
$Ti_3C_2T_x$/RGO	Foam	Freeze-drying	25-50	300 °C	[S14]
$Ti_3C_2T_x$/RGO	Aerogel	EDA crosslinking	10-90	85 °C	[S15]
$Ti_3C_2T_x$/RGO	Aerogel	Ascorbic acid reduction	25-75	90 °C	[S16]
$Ti_3C_2T_x$/RGO	Aerogel	Freeze-drying	25-75	RT	[S17]
$Ti_3C_2T_x$/RGO	Powder	Zinc powder reduction	90-95	RT	[S18]

Supplementary References

[S1] S. Zhao, H.B. Zhang, J.Q. Luo, Q.W. Wang, B. Xu et al., Highly electrically conductive three-dimensional $Ti_3C_2T_x$ MXene/reduced graphene oxide hybrid aerogels with excellent electromagnetic interference shielding performances. ACS Nano **12**(11), 11193-11202 (2018). https://doi.org/10.1021/acsnano.8b05739

[S2] X. Zhang, R. Lv, A. Wang, W. Guo, X. Liu et al., MXene aerogel scaffolds for high-rate lithium metal anodes. Angew. Chem. Int. Ed. **57**(46), 15028-15033 (2018). https://doi.org/10.1002/anie.201808714

[S3] Y. Ma, Y. Yue, H. Zhang, F. Cheng, W. Zhao et al., 3D synergistical MXene/reduced graphene oxide aerogel for a piezoresistive sensor. ACS Nano **12**(4), 3209-3216 (2018). https://doi.org/10.1021/acsnano.7b06909

[S4] Y. Yue, N. Liu, Y. Ma, S. Wang, W. Liu et al., Highly self-healable 3D microsupercapacitor with MXene–graphene composite aerogel. ACS Nano **12**(5), 4224-4232 (2018). https://doi.org/10.1021/acsnano.7b07528

[S5] W. Ma, H. Chen, S. Hou, Z. Huang, Y. Huang et al., Compressible highly stable 3D porous MXene/GO foam with a tunable high-performance stealth property in the terahertz band. ACS Appl. Mater. Interfaces **11**(28), 25369-25377 (2019). https://doi.org/10.1021/acsami.9b03406

[S6] Y. Chen, X. Xie, X. Xin, Z.R. Tang, Y.J. Xu, $Ti_3C_2T_x$-based three-dimensional hydrogel by a graphene oxide-assisted self-convergence process for enhanced photoredox catalysis. ACS Nano **13**(1), 295-304 (2019). https://doi.org/10.1021/acsnano.8b06136

[S7] T. Shang, Z. Lin, C. Qi, X. Liu, P. Li et al., 3D macroscopic architectures from self-assembled MXene hydrogels. Adv. Funct. Mater. **29**(33), 1903960 (2019). https://doi.org/10.1002/adfm.201903960

[S8] R. Butt, A.H. Siddique, S.W. Bokhari, S. Jiang, D. Lei et al., Niobium carbide/reduced graphene oxide hybrid porous aerogel as high capacity and long-life anode material for Li-ion batteries. Int. J. Energy Res. **43**(9), 4995-5003 (2019). https://doi.org/10.1002/er.4598

[S9] Z. Wang, N. Zhang, M. Yu, J. Liu, S. Wang et al., Boosting redox activity on MXene-induced multifunctional collaborative interface in high Li_2S loading cathode for high-energy Li-S and metallic Li-free rechargeable batteries. J. Energy Chem. **37**, 183-191 (2019). https://doi.org/10.1016/j.jechem.2019.03.012

[S10] Q. Wang, S. Wang, X. Guo, L. Ruan, N. Wei et al., MXene-reduced graphene oxide aerogel for aqueous zinc-ion hybrid supercapacitor with ultralong cycle life. Adv. Electron. Mater. **5**(12), 1900537 (2019). https://doi.org/10.1002/aelm.201900537

[S11] J. Song, X. Guo, J. Zhang, Y. Chen, C. Zhang et al., Rational design of free-standing 3D porous MXene/rGO hybrid aerogels as polysulfide reservoirs for high-energy lithium–sulfur batteries. J. Mater. Chem. A **7**(11), 6507-6513 (2019). http://dx.doi.org/10.1039/C9TA00212J

[S12] C. Yang, Q. Jiang, W. Li, H. He, L. Yang et al., Ultrafine Pt nanoparticle-decorated 3D hybrid architectures built from reduced graphene oxide and MXene nanosheets for methanol oxidation. Chem. Mater. **31**(22), 9277-9287 (2019). https://doi.org/10.1021/acs.chemmater.9b02115

[S13] Z. Lin, J. Liu, W. Peng, Y. Zhu, Y. Zhao et al., Highly stable 3D $Ti_3C_2T_x$ MXene-based foam architectures toward high-performance terahertz radiation shielding. ACS Nano **14**(2), 2109-2117 (2020). https://doi.org/10.1021/acsnano.9b08832

[S14] Z. Fan, D. Wang, Y. Yuan, Y. Wang, Z. Cheng et al., A lightweight and conductive MXene/graphene hybrid foam for superior electromagnetic interference shielding. Chem. Eng. J. **381**, 122696 (2020). https://doi.org/10.1016/j.cej.2019.122696

[S15] X. Zhao, L.M. Peng, C.Y. Tang, J.H. Pu, X.J. Zha et al., All-weather-available, continuous steam generation based on the synergistic photo-thermal and electro-thermal conversion by MXene-based aerogels. Mater. Horiz. **7**(3), 855-865 (2020). http://dx.doi.org/10.1039/C9MH01443H

[S16] D. Jiang, J. Zhang, S. Qin, Z. Wang, K.A.S. Usman et al., Superelastic $Ti_3C_2T_x$ MXene-based hybrid aerogels for compression-resilient devices. ACS Nano **15**(3), 5000-5010 (2021). https://doi.org/10.1021/acsnano.0c09959

[S17] L. Liang, Q. Li, X. Yan, Y. Feng, Y. Wang et al., Multifunctional magnetic $Ti_3C_2T_x$ MXene/graphene aerogel with superior electromagnetic wave absorption performance. ACS Nano **15**(4), 6622-6632 (2021). https://doi.org/10.1021/acsnano.0c09982

[S18] X. Yang, Q. Wang, K. Zhu, K. Ye, G. Wang et al., 3D porous oxidation-resistant MXene/graphene architectures induced by in situ zinc template toward high-performance supercapacitors. Adv. Funct. Mater. **31**(20), 2101087 (2021). https://doi.org/10.1002/adfm.202101087
